# 352例高龄肺癌术后心律失常的危险因素分析

**DOI:** 10.3779/j.issn.1009-3419.2011.03.11

**Published:** 2011-03-20

**Authors:** 嘉华 赵, 向阳 初, 志强 薛, 驰 徐

**Affiliations:** 100853 北京，中国人民解放军总医院胸外科 Department of Toracic Surgery, Chinese PLA General Hospital, Beijing 100853, China

**Keywords:** 肺肿瘤, 高龄, 心律失常, 危险因素, Lung neoplasms, Aged, Cardiac arrhythmia, Risk factors

## Abstract

**背景与目的:**

目前肺癌已经成为老年肿瘤患者中最常见的恶性肿瘤类型之一，而手术仍是其首选治疗手段，在老年肺癌患者术后并发症中，心律失常的发生率最高。本研究旨在分析70岁以上高龄肺癌患者术后早期心律失常的危险因素。

**方法:**

回顾性分析1997年7月-2008年5月在解放军总医院胸外科行原发性肺癌切除术的352例高龄患者中术后出现心律失常者的危险因素。单因素分析应用χ^2^检验，多因素分析应用*Logistic*回归进行统计。

**结果:**

352例高龄肺癌患者中，术后发生心律失常者71例，发生率为20.2%。经多因素分析发现，患者的年龄、合并心血管疾病史、术前心电图异常、一秒用力呼气容积（forced expiratory volume in one second, FEV1）、心脏射血分数（ejection fraction, EF）、手术方式的选择、手术时间与术后心律失常相关，且均为危险因素。

**结论:**

应注意预防高龄肺癌患者术后心律失常的发生。

## Abstract (en)

**Background and objective:**

Community is quickly turning into the aging period, and lung cancer has become more common in elderly patients with malignant tumor. At present, surgery remains the preferred therapeutic choice. However, it's worth noting that arrhythmia is a common complication during the perioperative period. Te aim of this study is to explore the risk factors of arrhythmia in lung cancer patients over the age of 70 years at perioperative periods.

**Methods:**

We retrospectively analyzed 352 patients who underwent lobectomy or pneumectomy in Chinese PLA General Hospital from July 1997 to May 2008. χ^2^ and *Logistic* regression were performed to analyze the clinical data.

**Results:**

Among 352 elderly patients with lung cancer, 71 cases accurred perioperative arrhythmias. Te occurrence rate was 20.2%. Te results of multivariate analysis showed that age, cardiovascular disease history, arrhythmia history, cardiac and pulmonary dysfunction before operation, pneumonectomy, operation time were the high risk factors of perioperative arrhythmia.

**Conclusion:**

More atention should be paid to prevent perioperative arrhythmia in the eldly lung cancer patients.

目前全球人口正迅速步入老龄化阶段，而年龄的增长与肺癌发病率密切相关，因此人口的老龄化已成为肺癌发病率增加的潜在因素^[1]^。虽然随着医疗技术水平的提高，肺癌的手术适应症不断扩大，也让越来越多的高龄肺癌患者可以接受手术治疗，但由于机体衰老导致的多器官多系统的生理储备功能下降，明显增加了术后相关并发症的发生率^[2]^。其中，心律失常是高龄肺癌术后最常见的并发症之一^[3, 4]^。如能明确高龄肺癌术后心律失常的危险因素，并对其给予早期干预，可为降低术后心律失常发生率、提高患者生存质量、加快患者术后恢复提供必要诊疗依据。本文将回顾性分析研究中国人民解放军总医院胸外科近年来高龄肺癌患者的病例资料，并探讨术后心律失常的主要危险因素。

## 1 材料与方法

### 1.1 研究对象

1997年7月-2008年5月在解放军总医院胸外科接受手术治疗的70岁以上肺癌患者352例，术后发生心律失常者71例。

### 1.2 观察指标

① 术前相关因素：患者的性别、年龄、吸烟史、术前是否合并有（高血压、冠心病、心梗及糖尿病）相关病史、术前心电图检查、术前心功能及肺功能检查；②手术相关因素：患者的病变部位、手术方式、淋巴结清扫、手术时间；③肿瘤相关因素：患者的术后病理分期及病理类型，淋巴结清扫有无阳性。

### 1.3 病例资料

高龄肺癌患者352例，男性270例，女性82例，年龄70岁-90岁[（73.5±2.9）岁]，其中≥75岁者108例， < 75岁者244例。有吸烟史174例，术前心电图检查异常者166例，有高血压史99例，有冠心病史35例，有心梗史11例，有糖尿病史34例。术前心功能检查心脏射血分数（ejection fraction, EF）≤50%者100例，肺功能检查一秒用力呼气容积（forced expiratory volume in one second, FEV1）≤1.5 L者130例。病变位于左肺者145例，位于右肺者207例。手术方式行肺叶切除273例，楔型切除64例，全肺切除11例，袖式肺叶切除4例。行淋巴结清扫288例，其中阳性淋巴结109例，手术时间 > 3 h者155例。病理类型：腺癌89例，鳞癌136例，支气管肺泡癌58例，小细胞癌12例，大细胞癌18例，腺鳞癌19例，混合型20例。肿瘤TNM分期依据术后病理结果按照国际抗癌联盟（Inernational Union Against Cancer, UICC）2009版分期法：其中Ⅰ期186例，Ⅱ期103例，Ⅲ期48例，Ⅳ期15例。

### 1.4 结果判定

患者术后采用多功能生命监护仪连续监护72 h以上，详细记录心律失常发生的时间、类型及治疗结果。本研究所追踪的心律失常类型主要分为：房性早搏、阵发性室上性心动过速、室性心动过速、窦性心动过速、心房纤颤、室性早搏（> 5次/分）、心室颤动、房室传导阻滞。对术后发生与术前同种类型心律失常者，因不能确定为何种因素所致，不计入术后影响因素分析。

### 1.5 统计学处理

采用SPSS 17.0软件进行数据统计，单因素资料分析应用χ^2^检验，多因素分析采用*Logistic*回归分析。*P* < 0.05为具有统计学差异。

## 2 结果

### 2.1 肺癌术后心律失常的发生情况

全组352例患者中术后71例发生心律失常（发生率为20.2%），其中窦性心动过速12例，房性早搏14例，阵发性室上性心动过速4例，心房纤颤31例，室性早搏（> 5次/分）8例，室性心动过速3例，心室颤动2例，房室传导阻滞8例，合并两种以上心律失常类型者11例。本组出现心律失常病例经积极抗心律失常药物治疗及体外电除颤后大部分转为正常心律，有9例心房纤颤患者经治疗后心室率明显减慢，但直至出院仍无法转复为窦性心律。全组患者均无死亡及严重并发症出现。

### 2.2 肺癌术后心律失常单因素分析

在术前相关因素分析中，患者的年龄、术前合并心血管（如高血压、冠心病、心梗及糖尿病）相关病史、术前检查心电图异常及心、肺功能异常者有统计学意义（表 1）；手术相关因素中，患者的手术切除方式及手术时间 > 3 h具有统计学意义（表 2）；在肿瘤相关因素中患者的术后病理类型有统计学意义（表 3）。在患者的手术切除方式中，全肺切除术后心律失常的发生率最高，而肺楔形切除术后心律失常的发生率则较低。其它因素如性别、吸烟史、肿瘤病变部位、淋巴结清扫及清扫之淋巴结有无阳性、肿瘤的TNM分期等则与高龄肺癌患者术后发生心律失常无相关性。

**1:** 纳入患者心律失常发生率的单因素分析（术前相关因素） Preoperative factors related to arrhythmia of all the patients analyzed by univariate analysis

Item	Total	Prevalence rate [*n* (%)]	*χ*^2^	*P*
Gender			0.744	0.107
Male	270	56 (20.7%)		
Female	82	15(18.3%)		
Age (year)			6.302	0.012
≥75	108	31 (28.7%)		
< 75	244	40 (16.4%)		
Smoking			0.085	0.437
Smoking	174	37 (21.3%)		
Non-smoking	178	34 (19.1%)		
History of angiocardiopathy			27.058	< 0.001
Accompanied	136	47 (34.5%)		
Not associated	216	24 (11.1%)		
Preoperative ECG			4.001	0.031
Abnormal	166	41 (24.7%)		
Normal	186	30 (16.1%)		
Preoperative EF			9.256	0.002
EF≤50%	100	31 (31.0%)		
EF > 50%	252	40 (15.9%)		
Preoperative FEV_1_			6.521	0.011
FEV1≤1.5 L	130	36 (27.7%)		
FEV1 > 1.5 L	222	35 (15.8%)		
ECG: electrocardiogram; EF: ejection fraction; FEV1: forced expiratory volume in one second.				

**2:** 纳入患者心律失常发生率的单因素分析（手术相关因素） Surgical factors related to arrhythmia of all the patients analyzed by univariate analysis

Item	Total	Prevalence rate [*n* (%)]	*χ*^2^	*P*
Lesion			1.023	0.312
Left lung	145	25 (17.2%)		
Right lung	207	46 (22.2%)		
Operation type			8.581	0.035
Pneumonectomy	11	6 (54.5%)		
Wedge resection	64	11 (17.2%)		
Lobectomy	273	53 (19.4%)		
Sleeve lobectomy	4	1 (25.0%)		
Lymph node-excision			0.001	0.547
Yes	288	58 (20.1%)		
No	64	13 (20.3%)		
Operation time			6.107	0.014
> 3 h	155	41 (26.5%)		
≤3 h	197	30 (15.2%)		

**3:** 纳入患者心律失常发生率的单因素分析（肿瘤相关因素） Tumor-associated factors related to arrhythmia of all the patients by univariate analysis

Item	Total	Prevalence rate [*n* (%)]	*χ*^2^	*P*
Lymph node-positive			1.504	0.229
Yes	109	26 (23.9%)		
No	179	32 (17.9%)		
Stage			3.616	0.306
Ⅰ	186	30 (16.1%)		
Ⅱ	103	23 (22.3%)		
Ⅲ	48	13 (27.1%)		
Ⅳ	15	3 (20.0%)		
Pathology			13.778	0.032
Adenocarcinoma	89	13 (14.6%)		
SCC	136	28 (20.6%)		
BAC	58	10 (17.2%)		
SCLC	12	1 (8.3%)		
LCLC	18	3 (16.7%)		
ASLC	19	8 (42.1%)		
Mixed	20	8 (40.0%)		
SCC: squamous cell carcinoma; BAC: bronchioloalveolar carcinoma; SCLC: small cell lung cancer; LCLC: large cell lung cancer; ASLC: adenosquamous carcinoma; Mixed: mixed lung cancer.				

### 2.3 肺癌术后心律失常多因素分析

将术前相关因素、手术相关因素及肿瘤相关因素中有统计学意义的各因素进行多因素分析可见（表 4）：本组中患者的年龄、既往合并有心血管系统疾病史、术前心肺功能异常者、手术方式的选择及手术时间的延长与术后心律失常相关（*P* < 0.05），且均为危险因素（*OR* > 1）。而肿瘤的病理类型与患者术后发生心律失常则无相关性（*P* > 0.05）。

**4:** 高龄肺癌术后心律失常的多因素分析 Multivariate analysis of arrhythmia in elderly after operation of lung cancer

	B	SE	Wals	*P*	OR	95%CI
Age≥75 years	1.782	0.413	18.593	< 0.001	5.943	2.644-13.361
History of angiocardiopathy	1.886	0.414	20.798	< 0.001	6.595	2.932-14.835
Preoperative ECG	1.366	0.470	8.437	0.004	3.920	1.559-9.855
Preoperative EF	1.319	0.464	8.065	0.005	3.740	1.505-9.293
Preoperative FEV1	1.878	0.413	20.706	< 0.001	6.538	2.912-14.679
Operation Type	1.853	0.416	19.885	< 0.001	6.381	2.826-14.411
Operation Time	0.007	0.003	6.987	0.008	1.007	1.002-1.012
Pathology	0.012	0.020	0.368	0.544	1.012	0.973-1.053

## 3 讨论

在高龄肺癌患者术后并发症中，心律失常的发生率占首位^[3]^。文献^[5]^报道其发生率约为25%，本组发生率为20.2%，低于相关文献报道，考虑可能与病例的选择及各自诊断标准不同有关。

在术前相关因素中，高龄及术前合并有心血管病史被认为是肺癌手术的高危因素^[6]^。而本组研究也印证了这一观点，患者的年龄≥75岁、合并心血管疾病史、术前心肺功能异常为高龄肺癌术后心律失常的危险因素。考虑是随着人体正常的老化过程，老年人的心脏血管会有一定的改变；无明显心血管疾病的正常老年人也存在着心肌细胞的增大和凋亡导致的心肌细胞数目的减少，窦房结的起搏细胞随着年龄的增长也会减少；当75岁时，窦房结仅留下10%的起搏细胞；静息状态下左心室早期充盈随着年龄的增长而下降，80岁时其下降率达50%；心脏的排出量、心肌对氧的利用率以及心肌收缩力均明显下降；其术后发生心律失常的概率明显升高^[7-10]^。尤其是患者术前心肺功能检查提示异常及合并有心律失常者，在肺切除术后，其心肺功能代偿能力进一步降低，更增加了心律失常的风险^[11]^。

在手术相关因素中，有文献^[12]^报道肺组织切除大小及范围与术后并发心律失常有相关性，因行肺切除时极有可能损伤到迷走和交感神经在主动脉弓与支气管分叉处构成的心丛，从而引起交感神经张力增高导致心律失常。本组研究中全肺切除并发心律失常的几率明显高于其它术式，考虑为全肺切除术由于创伤大、肺功能丧失多、肺血管床迅速减少为原来的一半、患侧成残腔后两侧压力不均衡而容易产生纵隔移位，导致肺循环阻力及心脏后负荷增大，这些因素均增加了术后心律失常的出现。楔形切除则相反，其术后并发心律失常几率是所有术式中最低的，考虑为楔形切除创伤小、分离结扎血管较少，减少了对肺门的牵拉与对心脏的挤压，降低了手术时间。有研究^[13]^表明肺楔形切除与肺叶切除对于原发性Ia期肺癌，肿瘤 < 3 cm者，其术后5年生存率无统计学差异，虽然整体生存率肺叶切除仍较楔形切除高。但笔者认为对于高龄患者，更应考虑到患者身体耐受手术情况及术后生存质量。因此对于早期高龄肺癌患者一般情况较差者，为预防术后心律失常及其它严重并发症的发生，进行肺楔形切除术也是一个不错的选择。

老年肺癌患者由于手术的创伤、麻醉药物对心脏的影响、疼痛、失血、缺氧、以及水、电解质和酸碱平衡失调，常可诱发老年人原有或潜在的心血管异常的患者出现心律失常，如处理不当可引起严重损害或突然死亡^[14]^。而本组研究中无患者出现死亡及其他严重并发症，考虑与中国人民解放军总医院胸外科所有高龄肺癌患者术前的全面评估及术后的及时处理有关。对于术前心肺功能较差，心电图异常及合并心血管疾病史者，给予全面检查及综合评估，必要时预防性给予抗心律失常药物。对于术前咳嗽、咳痰量较多者，预防性给予祛痰药物及应用抗生素，术后充分给氧、持续雾化吸入、保持呼吸道通畅及气道湿化。对于患者术后因乏力及疼痛导致咳痰较差，及时给予镇痛并鼓励其咳痰。也可通过按压患者胸骨上窝处刺激气道反应帮助其咳痰，必要时行纤维支气管镜吸痰，以预防因咳痰不力导致的肺不张及肺部感染。剧烈疼痛会导致交感神经兴奋，从而引起外周小动脉血管收缩、阻力增加，进一步增加了心脏的前负荷，诱发心律失常。因此对于术后疼痛剧烈者给予自控式微量止痛泵，此种方式止痛效果明显，在减轻患者术后痛苦的同时也大大降低了心律失常的发生率。

通过以上讨论可看出，周密的术前准备，严格控制手术指征，慎重选择高龄患者，有效治疗术前合并症，尽量纠正术前心律失常，改善心肺功能；术中操作避免过度牵拉肺门及挤压心脏，尽量缩短麻醉与手术时间。对于早期高龄肺癌患者尤其是肿瘤直径 < 3 cm者，应根据患者自身及手术耐受情况考虑选用楔形切除术；恰当的术后处理，积极鼓励咳嗽、咳痰，持续的低流量吸氧及雾化吸入。高危患者术后严密监测心肺功能，维持血压稳定和确保心肌供氧，纠正血流动力学紊乱，对降低高龄肺癌患者术后心律失常的发生率，保证其安全度过围手术期十分重要。
